# Maternal microbiota and fetal brain development: a multidisciplinary review of cognitive, neurological, and metabolic outcomes

**DOI:** 10.3389/fneur.2026.1778399

**Published:** 2026-09-29

**Authors:** Dmitry Satanovsky, Maria Beloyartseva, Pavel Kuznetsov, Nikita Savin, Anna Nesterenko, Katerina Vekilova

**Affiliations:** Ukrainian Society of Lifestyle Medicine, Kyiv, Ukraine

**Keywords:** autism spectrum disorder, fetal neurodevelopment, gut-brain axis, maternal immune activation, maternal microbiota, narrative review, neuroprotection, prenatal nutrition

## Abstract

**Objective:**

The prenatal period represents a critical window for the programming of the central nervous system. This multidisciplinary narrative review aims to synthesize current evidence regarding the maternal microbiota-gut-fetal brain axis, examining how maternal gut composition may influence fetal neurogenesis, synaptogenesis, and long-term neurobehavioural outcomes.

**Mechanism:**

We analyse three primary pathways of influence: the systemic translocation of neuroactive bacterial metabolites (specifically short-chain fatty acids), the modulation of the maternal immune system leading to maternal immune activation (MIA), and the endocrine regulation of the hypothalamic–pituitary–adrenal axis. These pathways are interconnected and their synergistic effects are considered throughout.

**Results:**

Neurological data, predominantly from animal models, suggest that maternal dysbiosis may disrupt microglial maturation and cortical lamination through epigenetic modifications mediated by butyrate and serotonin precursors. Endocrinological evidence highlights the role of the estrobolome and the bacterial conversion of glucocorticoids into neuroactive progestins. Obstetric and pediatric findings indicate associations between maternal microbiome composition, preterm birth risks, and the incidence of autism spectrum disorders and ADHD in offspring, though causal inference from human data remains limited. Nutritional analysis suggests that dietary fibers and Omega-3 fatty acids may act as modulators of this axis.

**Conclusion:**

The maternal microbiota is a potentially modifiable determinant of fetal neurological health. Microbiome-based interventions carry potential risks and require careful clinical evaluation. A comprehensive, individualized approach integrating neurology, endocrinology, obstetrics, and nutrition is required to develop evidence-based preventive strategies.

## Introduction: a neurological paradigm shift

Modern neurology and psychiatry are undergoing a paradigm shift regarding the origins of neurodevelopmental disorders. For decades, the placenta was viewed as a near-perfect barrier, and fetal brain development was considered a process determined almost exclusively by the genetic code and protected from the external environment. Accumulating evidence now invites us to reconsider this isolationist view (1–3). The prenatal period acts as a critical window of vulnerability and opportunity, where the maternal environment actively shapes the architecture of the developing central nervous system. At the center of this new understanding is the “maternal microbiota-gut-fetal brain axis” (4–6).

This axis represents a complex, bidirectional communication system. The trillions of microorganisms residing in the maternal gut function as a metabolically active organ, producing metabolites, neurotransmitters, and immune signals that can influence placental function or, in some cases, reach the fetal compartment (7–9). Perturbations in this system — termed dysbiosis (defined here as a reduction in microbial diversity or a compositional shift away from a health-associated community structure) — may not be silent. They may echo into the developing fetal brain, potentially altering gene expression in neurons, disrupting the formation of the blood–brain barrier, and modulating the maturation of microglia, the brain’s resident immune cells (9, 10).

Epidemiological and experimental data increasingly link maternal microbiome disruptions to a spectrum of neurodevelopmental conditions, ranging from autism spectrum disorder (ASD) and attention-deficit/hyperactivity disorder (ADHD) to subtle cognitive deficits and impaired stress resilience (11–13). It is important to note, however, that the majority of these associations derive from observational studies or animal models, and causal inference in humans remains limited. The task of ensuring optimal brain development may therefore begin with the metabolic and microbial health of the mother. This article presents a multidisciplinary narrative synthesis of this phenomenon, integrating perspectives from neurology, endocrinology, obstetrics, pediatrics, and nutrition to provide an overview of the mechanisms and clinical implications of the maternal microbiome (14).

A key premise of this review is that these disciplinary perspectives do not operate in isolation. The immune, endocrine, and metabolic pathways through which the maternal microbiome influences the fetal brain are interconnected: dysbiosis-induced immune activation can dysregulate the hypothalamic–pituitary–adrenal (HPA) axis; endocrine disruption through the estrobolome affects inflammatory tone; and nutritional factors modulate all of these pathways simultaneously. Figure 1 provides a schematic overview of these three converging pathways and their key intersection points. Where possible, we highlight these cross-disciplinary interactions rather than treating each pathway independently.

**Figure 1:**
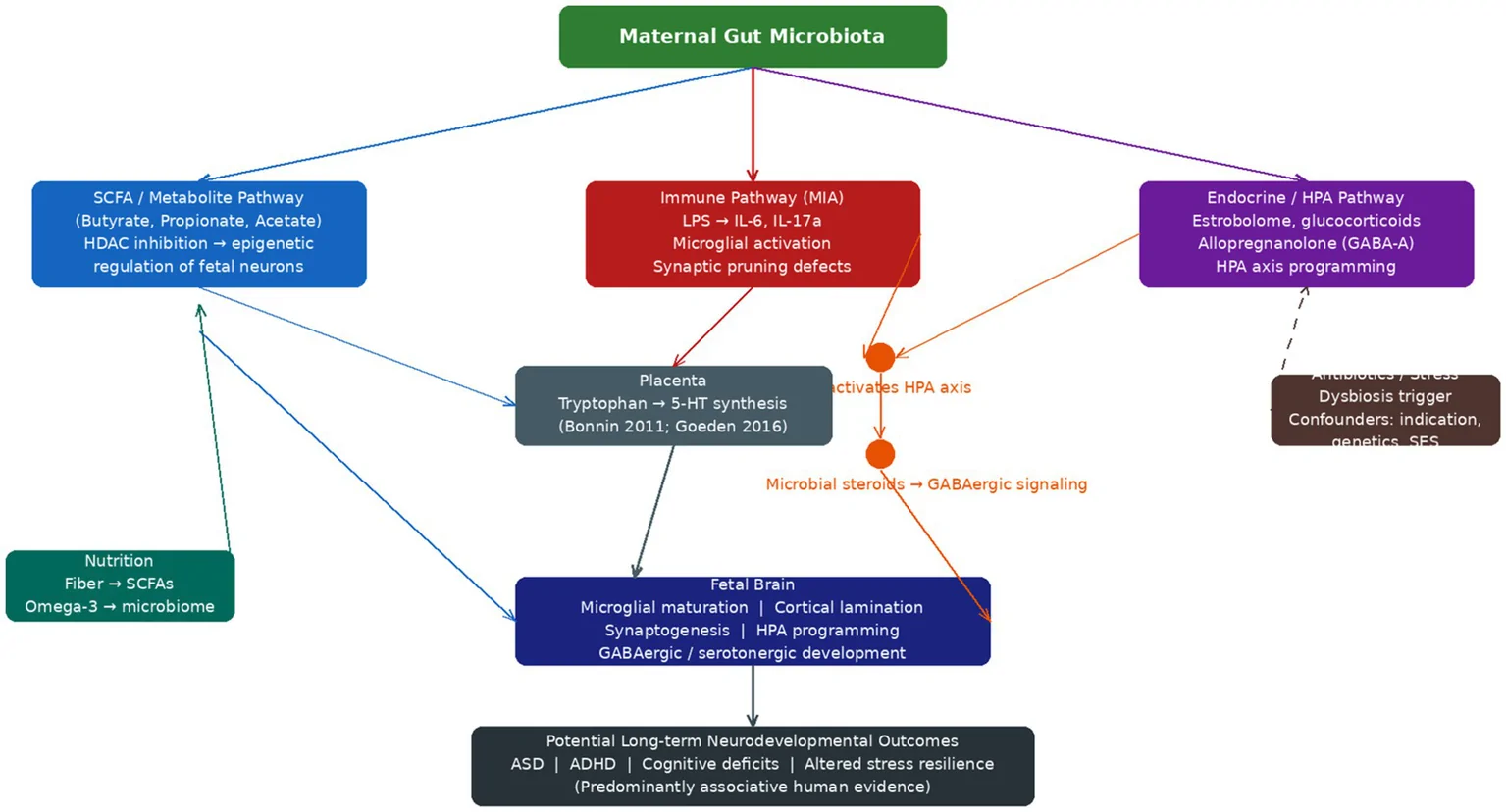
The maternal microbiota-gut-fetal brain axis: three converging pathways. Three primary pathways connect the maternal gut microbiota to the developing fetal brain: the SCFA/metabolite pathway (blue), the immune/MIA pathway (red), and the endocrine/HPA pathway (purple). Orange circles indicate key intersection points: MIA activating the maternal HPA axis, and microbial steroid metabolism feeding into GABAergic fetal brain signaling. Mechanistic routes shown are derived predominantly from animal models. Human evidence for most pathways is associative; evidence tiers are detailed in Table 1.

## Literature search strategy

This narrative review was conducted through a search of PubMed, Scopus, and Web of Science databases, covering publications from January 2015 to December 2025, with selective inclusion of seminal earlier works. Search terms included combinations of: “maternal microbiota,” “gut microbiome pregnancy,” “fetal neurodevelopment,” “maternal immune activation,” “short-chain fatty acids fetal brain,” “gut-brain axis prenatal,” “maternal dysbiosis offspring,” “prenatal nutrition microbiome,” “probiotic pregnancy randomized controlled trial,” “placental microbiome contamination.” Inclusion criteria: peer-reviewed original research, systematic reviews, and meta-analyses reporting on maternal microbiota composition or interventions in relation to fetal or offspring neurological, cognitive, or metabolic outcomes. Studies in animal models were included where they provided mechanistic evidence not yet available from human trials and are explicitly identified as such throughout the text. Negative findings, null results, and studies with attenuated associations were actively sought and included to provide a balanced synthesis. No formal risk-of-bias assessment was performed; this review therefore reflects a narrative rather than systematic synthesis.

## Neurological mechanisms of fetal programming

The influence of the maternal microbiota on the fetal brain is mediated through several distinct but interconnected pathways that together constitute the maternal microbiota-gut-fetal brain axis. These pathways do not operate independently: immune activation influences neuroendocrine function, metabolite production modulates immune tone, and nutritional factors affect all three simultaneously. Table 1 summarizes the primary pathways, their key mediators, the proposed fetal brain effects, and the evidence tier for each. Figure 1 illustrates the convergence of these pathways and their key intersection points.

**Table 1:** Summary of primary mechanistic pathways linking maternal microbiota to fetal brain development, with evidence tiering.

Pathway	Key mediator(s)	Fetal brain effect	Primary evidence tier
SCFA/metabolite	Butyrate (HDAC inhibitor); propionate; acetate	Epigenetic regulation of neuronal differentiation; myelination; BBB integrity	Murine models; limited human metabolomic data
Maternal immune activation (MIA)	IL-6; IL-17a; LPS translocation	Microglial dysregulation; synaptic pruning defects; cortical connectivity alterations	Murine models (19); human observational associations
Tryptophan/serotonin	Maternal tryptophan → placental 5-HT synthesis	Cortical neuron migration; hippocampal development	Murine + human placental data (21, 22)
HPA axis/glucocorticoids	Allopregnanolone; 11-oxygenated androgens	GABAergic programming; fetal stress response	Experimental (32, 33)
Estrobolome	Beta-glucuronidase; beta-glucosidase	Estrogen bioavailability; neuroendocrine programming	Human microbiome studies (28, 29)
Metabolic/GDM pathway	GLP-1; PYY; insulin resistance	Adverse fetal metabolic milieu; neurodevelopmental risk	Human observational (26, 27)

BBB, blood–brain barrier; 5-HT, serotonin; HPA, hypothalamic–pituitary–adrenal; GDM, gestational diabetes mellitus; LPS, lipopolysaccharides.

The first and perhaps most direct pathway involves the production of neuroactive metabolites. The gut microbiota is a prolific producer of short-chain fatty acids (SCFAs), primarily butyrate, propionate, and acetate, derived from the fermentation of dietary fibers (15, 16). These molecules enter the maternal circulation and have been detected in fetal tissues in animal studies, with their capacity to reach the human fetal brain remaining to be fully established (9, 10).

From a neurological perspective, butyrate is particularly significant because it functions as a histone deacetylase (HDAC) inhibitor. In murine models, bacterial butyrate exerts a direct epigenetic influence on fetal neurons, increasing the acetylation of histones and thereby promoting the transcription of genes essential for neuronal differentiation and plasticity (1, 6). Animal studies further indicate that a maternal deficit in SCFA-producing bacteria is associated with impaired myelination and deficits in the expression of tight junction proteins, which are critical for the integrity of the developing blood–brain barrier (10, 15). Whether these mechanisms translate directly to human fetal neurodevelopment remains an active area of investigation.

The second pathway is immunological. The composition of the maternal gut microbiota influences the maternal immune system. Dysbiosis may lead to a compromised intestinal barrier — colloquially termed “leaky gut,” characterized by increased epithelial permeability and bacterial endotoxin translocation — resulting in the entry of lipopolysaccharides into the systemic circulation and a state of chronic, low-grade inflammation. This systemic inflammation can trigger Maternal Immune Activation (MIA) (17, 18). During MIA, elevated levels of proinflammatory cytokines, particularly interleukin-6 (IL-6) and interleukin-17a (IL-17a), interact with the placental barrier (18, 19). Both murine and limited human data suggest that these immune signals can contribute to the aberrant activation of fetal microglia. Since microglia are responsible for synaptic pruning — the process of eliminating excess neural connections to refine brain circuits — their dysfunction may contribute to hyper-connectivity or hypo-connectivity in critical brain regions, features observed in conditions such as autism and schizophrenia (14, 17, 19). Critically, the immune and endocrine pathways intersect: MIA activates the maternal HPA axis, elevating glucocorticoids whose subsequent microbial metabolism may further alter the fetal hormonal environment (Figure 1).

The third major pathway involves the regulation of neurotransmitter precursors. Tryptophan metabolism is substantially influenced by the gut microbiota. Tryptophan is the essential precursor for serotonin (5-HT) (20). A critical clarification is required here: the predominant mechanism by which serotonin reaches the fetal forebrain is not the direct transfer of maternal serotonin across the placenta. Rather, the placenta itself synthesizes serotonin *de novo* from maternally-derived tryptophan, and this placenta-derived serotonin is the primary source for the developing fetal forebrain during early embryogenesis, before the fetal raphe nuclei are capable of independent synthesis (21, 22). Serotonin in this stage acts not merely as a neurotransmitter but as a vital trophic factor guiding the migration of excitatory neurons into the cerebral cortex and hippocampus (21, 22). Consequently, maternal gut dysbiosis that depletes tryptophan availability may impair placental serotonin synthesis, with downstream effects on cortical neuron migration. Experimental evidence suggests this pathway may contribute to structural anomalies in cortical lamination and hippocampal volume (1, 12). These data derive predominantly from murine models; human evidence for this specific pathway remains limited to associative studies.

## Endocrine and metabolic regulation of the axis

The interaction between the maternal host and her microbiome extends into the endocrine system, forming a regulatory network that is deeply interwoven with the immunological and neurological pathways described above. The total genome of the gut microbiota contains approximately 3 million genes — roughly 150 times the size of the human genome — providing substantial catalytic capacity for these cross-system interactions, though this figure represents an order-of-magnitude estimate based on metagenome-wide association studies (8, 23).

### Evidence from human pregnancy studies

Pregnancy is accompanied by physiological shifts in gut microbial composition, including reported increases in genera such as Bifidobacterium and Akkermansia, as well as certain members of the Firmicutes phylum (24, 25). These adaptive changes are thought to promote energy harvest and storage during pregnancy to support fetal growth. Such microbial alterations have been associated with increased production of SCFAs, which activate G-protein-coupled receptors, particularly GPR41 and GPR43. Activation of these receptors has been implicated in the regulation of maternal energy balance, lipid metabolism, and insulin sensitivity (15, 26).

In some pregnancies, disruption of these adaptive microbial changes may contribute to dysbiosis. Reduced microbial diversity and alterations in specific bacterial taxa have been associated with metabolic complications, including gestational diabetes mellitus (GDM). The resulting hyperglycemia and insulin resistance may create an adverse metabolic environment that could contribute to altered fetal brain development (26, 27). These associations are observational and subject to substantial confounding by maternal diet, pre-pregnancy BMI, and socioeconomic factors.

The maternal gut microbiome may also influence endocrine homeostasis through the estrobolome — defined as the collection of microbial genes involved in estrogen metabolism via beta-glucuronidase and beta-glucosidase activity. Certain gut bacteria express these enzymes to deconjugate estrogens in the intestine, allowing their reabsorption into the circulation, thereby influencing systemic estrogen bioavailability and maternal endocrine regulation (28, 29).

Beyond steroid hormones, the microbiome may modulate non-steroid hormones, including glucagon-like peptide-1 (GLP-1), peptide YY (PYY), and ghrelin through interactions with enteroendocrine cells (30). Disruption of these signaling pathways has been associated with metabolic disorders such as obesity and type 2 diabetes, which are recognized risk factors for adverse neurodevelopmental outcomes in offspring (8, 27).

### Mechanistic insights from experimental studies

Experimental studies suggest that the gut microbiota participates in steroid hormone metabolism through several microbial enzymatic pathways. Bacteria such as Clostridium steroidoreducens have been reported to utilize reductive pathways that convert glucocorticoids into tetrahydrosteroid metabolites (31, 32). Gut commensals including *Gordonibacter pamelaeae* and *Eggerthella lenta* have been reported to convert glucocorticoids into progestin-like metabolites via 21-dehydroxylation, potentially contributing to increased levels of neuroactive steroids such as allopregnanolone, a potent modulator of GABA-A receptors (33). Experimental evidence also suggests that *Ruminococcus gnavus* may metabolize cortisol into 11-oxygenated androgen metabolites (31, 32).

These findings illustrate how the immune-endocrine-neural pathways converge: stress-induced glucocorticoid elevation feeds into microbial steroid transformation, which in turn modulates GABAergic signaling in the fetal brain — a plausible mechanistic chain linking maternal psychological stress, gut dysbiosis, and altered fetal neurodevelopment through a unified pathway (34). However, these steroid-transforming pathways derive from preclinical studies, and their quantitative relevance during human pregnancy remains to be established.

## The reproductive tract microbiota: relevance to pregnancy outcomes and initial colonization

The reproductive tract microbiota connects to the core theme of this review through two distinct mechanisms: first, by influencing pregnancy maintenance and thereby the intrauterine environment in which fetal brain development occurs; and second, by determining the initial microbial colonization of the neonate, which shapes postnatal neuro-immune programming. The vaginal mucosa is typically dominated by Lactobacillus species such as *L. crispatus*; lactic acid production maintains a low pH that inhibits pathogen overgrowth (35, 36). Dysbiosis — characterized by Lactobacillus depletion and anaerobic overgrowth similar to bacterial vaginosis — is associated with implantation failure in IVF cycles and early pregnancy loss (37). Sustained dysbiosis with pathogens such as Gardnerella or Ureaplasma triggers proinflammatory cytokine responses in the cervicovaginal fluid, associated with an increased risk of preterm birth (36, 38). Preterm birth is itself an independent risk factor for neurodevelopmental impairment, creating a direct pathway from vaginal dysbiosis to adverse neurological outcomes through obstetric mediation.

### The placental microbiome: current evidence and methodological considerations

A significant controversy in modern obstetrics concerns the existence of a “placental microbiome.” Earlier sequencing studies identified bacterial DNA signatures in placental tissue; however, subsequent methodologically rigorous investigations — employing negative controls, reagent contamination profiling, and batch-effect correction — demonstrated that these signals were largely indistinguishable from background contamination and laboratory reagent microbiomes (39, 40).

Two independent systematic reviews and re-analyses of 16S rRNA sequencing datasets (41, 42) reached the same conclusion: placental microbiome signals correlated with mode of delivery rather than gestational status, consistent with a contamination origin. Using shotgun metagenomic sequencing — substantially more resistant to contamination than 16S rRNA approaches — Gschwind et al. (43) found bacteria only outside the placental tissue itself. Olaniyi et al. (44), examining 120 placental samples collected under strict sterile conditions during elective caesarean section, detected only very low bacterial abundances comprising skin commensals and environmental organisms.

High-quality null findings currently represent the strongest available evidence and support the traditional view of a sterile intrauterine environment in healthy term pregnancies (40). What remains genuinely uncertain is whether low-biomass transient microbial communities may exist under specific pathological conditions such as chorioamnionitis, or in early gestation, without constituting a stable resident microbiome. Claims of a functional placental microbiome in healthy term pregnancies should not be accepted without substantially more rigorous contamination controls.

### Neonatal colonization dynamics

Recent longitudinal sequencing of mother-infant pairs indicates that the maternal gut microbiota at 32 weeks of gestation is the primary contributor to the meconium microbiota of vaginally delivered infants, with a greater contribution than vaginal flora (45). This finding derives from a single 2025 study, and multiple earlier investigations have reached different conclusions regarding the primary source of neonatal colonization. The mechanistic basis — whether reflecting perinatal transmission during delivery, methodological factors, or other pathways — remains to be established and should not be interpreted as evidence of intrauterine colonization in healthy pregnancies. Regardless, the pattern of maternal-to-neonatal microbial transmission is clinically significant, as initial colonization instructs the developing neonatal immune system (17, 46).

## Pediatric implications: from DOHaD to clinical practice

The DOHaD (Developmental Origins of Health and Disease) framework provides the clinical context for interpreting the associations reviewed above. A critical and explicit distinction must be maintained throughout: the evidence linking maternal microbiota to offspring neurodevelopment is predominantly associative in humans, whereas mechanistic causality is more robustly demonstrated in animal models.

Human evidence supports associations between perinatal exposures that perturb the microbiome and later neurodevelopmental outcomes. For antibiotic exposure, the primary meta-analytic evidence derives from Tao et al. [Front Neurol 2022 (47)], a systematic review of 15 studies reporting prenatal antibiotic exposure was associated with increased risk of ADHD (OR 1.14; 95% CI 1.13–1.15) and, in prospective cohort subgroup analyses, ASD (OR 1.17; 95% CI 1.03–1.31). These signals were attenuated or absent in sibling-controlled analyses in other studies, and overall certainty remains very low, reflecting substantial familial and indication-related confounding. Maternal infection itself — the indication for antibiotic treatment — is an independent risk factor for neurodevelopmental disorders through MIA pathways. Disentangling antibiotic-induced dysbiosis from the underlying infection remains methodologically challenging, and causal inference is not currently supported by the available human data (11, 12).

A second line of evidence concerns the predictive value of the maternal microbiome. In a large human cohort, the maternal prenatal gut microbiome was more strongly associated with infant neurodevelopmental scores at one year of age (ASQ-based measures) than the infant gut microbiome at the same age, consistent with a prenatal programming effect (13). These findings remain associative, are based on a single cohort, and are not suitable for individual-level clinical prediction. Sex-specific differences are also observed; male fetuses appear more susceptible to the effects of maternal immune activation and dysbiosis, which may partly explain the higher prevalence of ASD and ADHD in boys (14, 19).

Regarding racial, geographic, and genetic influences: the composition of the maternal gut microbiota varies substantially across populations, influenced by habitual diet, geographic region, genetic background, and socioeconomic factors. Studies conducted predominantly in high-income Western populations may not generalize to other settings where microbiome composition, dietary patterns, and baseline rates of dysbiosis differ markedly. This represents a significant gap in the current evidence base.

Clinical recommendations must remain preventive and safety-calibrated. Antibiotic stewardship in pregnancy and early childhood remains essential: treatment should not be withheld when clinically indicated, but unnecessary exposure should be minimized (11). The “microbial inheritance” — the ensemble of microorganisms vertically transmitted from mother to offspring perinatally — may influence the metabolic and neurological trajectory of the child in the long term, though the magnitude and modifiability of this effect in humans requires further investigation (46).

## Nutritional modulation of the maternal-fetal axis

Nutritional factors represent the most accessible modifiable influence on maternal gut microbial composition. A critical distinction must be maintained between evidence that dietary interventions modify the maternal microbiome (reasonably established) and evidence that such microbiome modification improves fetal neurodevelopmental outcomes (largely unestablished in humans).

### Dietary fiber

Dietary fiber is the primary fermentable substrate for butyrate-producing gut bacteria. An intake of approximately 28 grams per day — consistent with dietary reference values for pregnancy established by the Institute of Medicine (IOM) and the European Food Safety Authority (EFSA) (48) — is associated with a microbiome composition more favorable to SCFA production (15). Soluble fibers (e.g., inulin, pectin, beta-glucan, found in oats, legumes, and fruit) are preferentially fermented by Bifidobacterium and Faecalibacterium and produce higher butyrate yields. Insoluble fibers (e.g., cellulose in vegetables and whole grains) contribute to transit time and stool bulk but produce fewer SCFAs. Fiber intake in the first and second trimesters is most relevant to microbiome establishment; by the third trimester, microbial diversity has typically declined physiologically and is less responsive to dietary modification (25). Tolerability varies: rapid increases in fiber intake may cause bloating and flatulence, and individualized dietary guidance is preferable to uniform targets.

### Omega-3 polyunsaturated fatty acids

DHA and EPA have established roles in fetal brain structural development independent of microbiome effects. Their interaction with the maternal microbiome appears bidirectional: Omega-3 s increase the abundance of Bifidobacterium and Lactobacillus while attenuating gut inflammation, and a healthy microbiome may support efficient absorption of these fatty acids (3, 49). The WHO recommends a minimum of 200 mg DHA per day during pregnancy, achievable through 1–2 portions of low-mercury oily fish per week or algae-derived supplementation. Whether Omega-3 supplementation improves neurodevelopmental outcomes via microbiome-mediated mechanisms specifically has not been established in human trials.

### Fermented foods and probiotic supplementation

Fermented foods (yogurt, kefir, fermented vegetables) provide transient exposure to beneficial microbes and their metabolites. Preliminary observational evidence associates their consumption with lower maternal cortisol levels and reduced inflammatory markers during pregnancy; however, these effects have not been consistently replicated in adequately powered randomized trials, and downstream effects on fetal neurodevelopment remain unestablished (34, 36).

Probiotic supplementation during pregnancy has been evaluated primarily for metabolic and obstetric endpoints. *Lactobacillus rhamnosus* GG has the most robust safety data in pregnancy (36). *Lactobacillus reuteri* RC-14 and *Lactobacillus rhamnosus* GR-1 have been studied for vaginal dysbiosis. *Bifidobacterium longum* and *Lactobacillus acidophilus* combinations have been evaluated for GDM prevention with modest effects on glycemic control. Critically, no large-scale randomized controlled trial has demonstrated that probiotic supplementation during pregnancy improves neurodevelopmental outcomes in offspring measured at or beyond two years of age. Multiple trials examining probiotic effects on infant allergy, eczema, and colic have shown null or inconsistent results for closely related immune endpoints.

Potential adverse effects must be acknowledged. Although serious adverse events are rare in immunocompetent individuals, infection risk in immunocompromised pregnant women, women with small intestinal bacterial overgrowth, or those receiving immunosuppressive therapy is a clinical concern. Probiotic supplementation should not be recommended universally but considered on an individualized basis.

### Other nutritional factors and practical guidance

Adequate intake of choline (450 mg/day during pregnancy per IOM), folate (400–600 mcg/day), and iodine (220 mcg/day) supports fetal neurodevelopment through pathways that interact with but are distinct from microbiome-mediated mechanisms. Minimization of ultra-processed foods and excessive saturated fat intake is associated with reduced gut inflammation and greater microbial diversity (49). All nutritional guidance should be individualized in clinical practice, accounting for pre-existing dietary patterns, cultural food practices, metabolic status, and gestational trimester.

## Limitations

Several limitations of the current evidence base warrant explicit acknowledgment. First, a substantial proportion of mechanistic insights derives from murine models. Mice differ from humans in gut microbiota composition, placental architecture, gestational immunology, and neurodevelopmental timelines; findings from germ-free or antibiotic-treated mouse models should be interpreted with particular caution.

Second, human observational studies are subject to significant confounding. Maternal diet, socioeconomic status, antibiotic use, mode of delivery, gestational age, infant feeding practices, geographic region, ethnicity, and genetic background are all intercorrelated determinants of both microbiome composition and neurodevelopmental outcomes, and are rarely fully adjusted for simultaneously.

Third, microbiome research is methodologically heterogeneous. Differences in DNA extraction protocols, sequencing platforms (16S rRNA vs. shotgun metagenomics), variable regions amplified, reference databases, and contamination-control practices limit cross-study comparability and may generate spurious findings, particularly in low-biomass compartments such as the placenta or amniotic fluid.

Fourth, most human studies rely on indirect or proxy outcomes. Direct measures of fetal brain structure or function in relation to maternal microbiota composition in humans remain scarce.

Fifth, important negative findings exist that constrain the strength of conclusions. Multiple randomized controlled trials of probiotic and prebiotic interventions during pregnancy have not demonstrated improvements in neurodevelopmental outcomes in offspring. The absence of positive interventional evidence should temper enthusiasm for translating observational associations into clinical practice recommendations.

Sixth, publication bias toward positive findings may overrepresent associations in the current literature.

Seventh, the current evidence base derives disproportionately from high-income Western populations. Racial, geographic, and genetic influences on maternal microbiota composition and the effectiveness of microbiome-targeted interventions remain insufficiently characterized.

## Future perspectives

Several research priorities emerge from this review. Adequately powered randomized controlled trials of targeted probiotic and prebiotic interventions during pregnancy, with pre-specified neurodevelopmental outcomes measured in offspring beyond two years of age, are the most important unmet need in this field. Such trials must include active placebo controls, long-term safety monitoring, and stratification by baseline maternal microbiome composition.

Multi-omic longitudinal cohort studies integrating maternal metagenomics, metabolomics, immunological profiling, and fetal neuroimaging would allow mechanistic hypotheses generated in animal models to be tested systematically in humans. Methodological standardization — including universal contamination-control protocols, reference databases stratified by gestational age and geographic region, and adoption of shotgun metagenomics as a standard over 16S rRNA in low-biomass compartments — is a prerequisite for reliable cross-study comparison.

Research must also address population diversity. Studies in geographically and ethnically diverse cohorts are necessary to determine whether associations observed in Western populations generalize. Investigation of sex-specific vulnerability windows and epigenetic mechanisms of microbiota-brain programming may further refine understanding of differential susceptibility. The potential of microbiome-targeted therapeutics — including dietary precision medicine and psychobiotic interventions for prenatal stress — represents a clinically promising but currently investigational frontier requiring adequately powered safety and efficacy trials before clinical translation.

## Conclusion

The evidence synthesized in this review suggests that the maternal microbiota is an active participant in shaping the prenatal neurodevelopmental environment, operating through interconnected immune, endocrine, and metabolic pathways rather than through isolated mechanisms (Figure 1). Through the generation of epigenetic metabolites such as butyrate, the modulation of maternal immune tone, the regulation of the hormonal milieu, and the placental synthesis of serotonin from maternal tryptophan, the gut microbiome may exert a meaningful influence on the developing brain (4–6, 21, 22).

Two important qualifications frame the clinical interpretation of this evidence. First, the majority of mechanistic insights derive from animal models and have not been confirmed in adequately powered human interventional studies. Second, microbiome-based interventions during pregnancy carry potential risks — including infection risk in vulnerable populations and the possibility of unintended ecological disruption — that require careful clinical evaluation before implementation.

Future research must move from observational associations to causal mechanisms and interventional trials in humans, with attention to population diversity, methodological rigor, and long-term safety. The current body of knowledge supports a clinical approach that prioritizes established preventive measures — evidence-based nutrition, appropriate antibiotic stewardship, and stress management — as part of comprehensive prenatal care, while approaching microbiome-specific interventions with the caution that the evidence base currently warrants (14).
